# Experiences of patients, family and professional caregivers with Integrated Palliative Care in Europe: protocol for an international, multicenter, prospective, mixed method study

**DOI:** 10.1186/1472-684X-13-52

**Published:** 2014-11-21

**Authors:** Marlieke van der Eerden, Agnes Csikos, Csilla Busa, Sean Hughes, Lukas Radbruch, Johan Menten, Jeroen Hasselaar, Marieke Groot

**Affiliations:** Department of Anaesthesiology, Pain and Palliative Care, Radboud University Medical Center, Nijmegen, The Netherlands; Department of Family Medicine, University of Pecs Medical School (UP), Pécs, Hungary; Division of Health Research, International Observatory on End of Life Care, Lancaster university, Lancaster, UK; Klinik für Palliativmedizin, Universitätsklinikum Bonn, Bonn, Germany; Radiation Oncology Department, University Hospital Leuven, Leuven, Belgium

**Keywords:** Palliative care, Integrated care, Patient experiences, Mixed method

## Abstract

**Background:**

The number of people living with advanced cancer and chronic disease has increased worldwide. Many of these patients could benefit from palliative care, focusing on optimising the quality of life of patients and their families facing problems resulting from life-threatening diseases. However, fragmentation and discontinuity of palliative care services often result in suboptimal palliative care. In order to overcome these problems, models using an integrated care approach are increasingly advocated in palliative care services. Although several models and definitions of Integrated Palliative Care (IPC) have been developed, the effects of integrated care are still under-investigated. Knowledge of the key components that constitute successful palliative care integration is still lacking. This mixed methods study will examine the experiences of patients, family caregivers and professional caregivers in order to provide insight into the mechanisms that constitute successful palliative care integration.

**Methods/Design:**

Prospective multiple embedded case study. Three to five integrated palliative care initiatives will be selected in Belgium, Germany, Hungary, The Netherlands and the United Kingdom. Data collection will involve Social Network Analysis (SNA), a patient diary, semi-structured interviews, and questionnaires: Palliative care Outcome Scale (POS), Canhelp Lite, Caregiver Reaction Assessment (CRA). Patients and family caregivers will be followed in 4 consecutive contact moments over 3 months. The diary will be kept weekly by patients. One focus group per initiative will be conducted with professional caregivers. Interviews and focus groups will be tape recorded, transcribed and qualitatively analysed using NVivo 10. SPSS Statistics 20 will be used for statistical analysis.

**Discussion:**

This study will provide valuable knowledge about barriers, opportunities and good practices in palliative care integration in the selected initiatives across countries. This knowledge can be used in the benchmark of integrated palliative care initiatives across Europe. It will add to the scientific evidence for IPC services internationally and will contribute to improvements in the quality of care and the quality of living and dying of severely ill patients and their relatives in Europe.

**Electronic supplementary material:**

The online version of this article (doi:10.1186/1472-684X-13-52) contains supplementary material, which is available to authorized users.

## Background

The number of people living with advanced cancer and chronic non-malignant disease has increased worldwide [1]. Patients often suffer from symptoms related to their illness and experience reduced quality of life [2, 3]. Many of these patients could benefit from palliative care, focusing on optimising the quality of life of patients and their families facing problems resulting from life-threatening diseases [4]. Literature has shown that palliative care has a positive impact on the quality of life of patients with advanced cancer and advanced chronic disease at lower costs [5, 6]. However, fragmentation and discontinuity of palliative care services in Europe often result in suboptimal palliative care [7]. Many patients receive palliative care in a very late stage of their illness or not at all. This applies even more to patients with non-malignant disease, such as patients with Chronic Obstructive Pulmonary Disease (COPD) and Chronic Heart Failure (CHF), compared to patients with malignant disease [8–10]. As a result, many patients lack adequate control or relief of symptoms to maintain quality of life. Moreover, these patients often visit out-of-hours services due to uncontrolled pain and other symptoms, or experience hospital admissions during the last phase of life [11, 12]. Consequently many patients are not able to die at their preferred place of death [3, 13].

In order to overcome these problems, models using an integrated care approach are increasingly advocated in palliative care. These endeavours focus on the integration of palliative care either early in a certain disease trajectory and/or in the organisation of care, by collaboration and consultation with experienced (palliative) care services and specialists [14, 15]. Initiatives using an integrated palliative care (IPC) approach have shown promising results, reducing fragmentation and enhancing continuity of palliative care [14]. Greer [5] and Zimmermann [16] showed that integration of palliative care into standard care for patients with malignant disease, could positively affect outcomes such as quality of life, quality of care and symptoms. Epiphaniou [17] showed that integration of palliative care into the organisation of care, by means of improved coordination and communication between all primary and secondary caregivers involved in the palliative care network of patients with lung cancer and COPD, enhanced continuity of care. Although several models and definitions of IPC have been developed, the effects of integrated care are still under-investigated [18, 19]. Promising results are mainly based on the evaluation of individual services, using retrospective or cross-sectional data [8, 20, 21]. Knowledge of the key components that constitute successful palliative care integration is still lacking [22, 23].

In order to address this knowledge gap, the EU-funded (FP7) collaborative research project “*Patient-centred integrated palliative care pathways in advanced cancer and chronic disease”* (InSup-C) was planned. The aim of the overarching study is to identify best or promising practices in IPC across Europe. A central component of the project is a prospective mixed methods cohort study that will be carried out with patients and their caregivers receiving palliative care. This mixed methods study will examine the experiences of patients, family caregivers and professional caregivers with palliative care provision and will provide insight into the mechanisms that constitute successful palliative care integration. We expect that this knowledge will contribute to the improvement and implementation of IPC across Europe.

The research question addressed in this study is:

*How do patients with advanced cancer, COPD and CHF, their family and professional caregivers experience care provision in a range of IPC initiatives in five European countries?*

This question will be explored by an examination of what care is provided by whom and to what extend caregivers work together to provide patient-centred, continuous care. Important aspects of this exploration also entail whether the needs, problems and expectations of patients and family caregivers are met, and how relationships between patients/family caregivers and professional caregivers are experienced. As family caregivers are often closely involved in palliative care provision, their perspectives on caring for the patient will also be explored. Subsidiary questions emerging from the research question are:

1. How is the care network of the patient organised with respect to the type, properties and quality of relationships between patients and family/professional caregivers?
2. What opinions do patients and family and professional caregivers have on the continuity and quality of care provided?
3. How do patients rate their symptoms and quality of life?
4. How do family caregivers rate their burden or reward of care giving?

The objective of this paper is to present the protocol of this patient and caregivers study, including a detailed description of the study design and the methodological approach. The methodology described in this paper will also serve as a reference for future publications about the study.

## Methods/Design

### Study design

**Figure 1:**
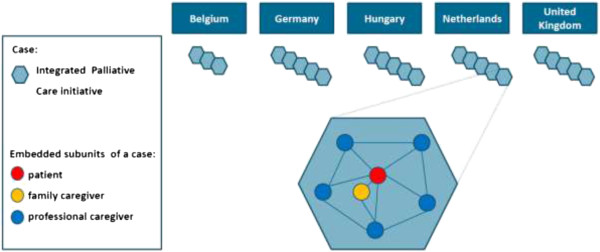
**Multiple embedded case study design of our study about the experiences of patients, family and professional caregivers with IPC in Europe.**

This study uses a prospective multiple embedded case study design [24]. This design enables us to examine the quality of care of a range of IPC initiatives in-depth and over time, as it is experienced in daily care giving practice. This design also allows us to explore the embedded subunits of multiple cases in order to understand more about the case itself. In this study the cases are IPC initiatives (see Figure 1). The embedded subunits are patients, family caregivers and professional caregivers and their experiences with care provision in the initiatives. Detailed analysis of these embedded subunits includes: the organisation of the patient’s care network and relationships with and collaboration between professional caregivers in this network, perceived quality of care, quality of life and symptoms of patients, perceived burden and reward of care giving of family caregivers. The multiple case study design also allows for comparison between IPC initiatives, each one with its own organisation and set-up (roles, responsibilities, relationships). In order to enable comparison the data collection methods for patients, family caregivers and professional caregivers will be the same for all IPC initiatives.

### Selection of IPC initiatives (cases)

The study will be conducted in five European countries; Belgium (Flanders region), Germany, Hungary, The Netherlands and the United Kingdom. In each country, three (Belgium) or five (the other countries) IPC initiatives will be recruited to the study. It is a novelty that in palliative care research patients and (family) caregivers’ views will be investigated at this large European scale. As integrated palliative care is an upcoming and under-investigated field, there is no theoretical framework or prevailing standard on which we can base the number and selection of cases (IPC initiatives). We expect that three-five initiatives per country and 23 in total will be enough to generate insight in how current IPC initiatives vary in service provision (e.g. diagnostic group(s), organisational structure, caregivers and settings that are involved, etc.) and what this means for patients’ and caregivers’ perspectives on the (quality of) service provision and its barriers and benefits. Although this number may not be enough to reach full saturation, we need to take into account the reality of inclusion of a rather vulnerable patient group which will demand large efforts in time and resources in the project team.

As there was no unanimously agreed definition of IPC beforehand, the project team formulated a working definition. This was based on the literature [25–29] and on discussions in several project team meetings. The final definition is as follows: “*Integrated palliative care involves bringing together administrative, organisational, clinical and service aspects in order to realise continuity of care between all actors involved in the care network of patients receiving palliative care. It aims to achieve quality of life and a well-supported dying process for the patient and the family in collaboration with all the caregivers, paid and unpaid”*. Using this definition and the criteria below, which derived from the definition, local initiatives in the five countries will be identified, examined, and selected for inclusion. Experts in palliative and integrated care across the participating nations will be consulted in the identification process.

### Selection criteria for IPC initiatives

1. the initiative is an established local palliative care collaboration;
2. the collaboration must contain at least two different organisations;
3. a hospital can be part of that collaboration;
4. collaborating healthcare professionals must provide direct patient care (not only an advisory function);
5. the collaboration has a multidisciplinary background (professionals of different professions must be involved, e.g. physician (specialist, GP), nurse (specialist), social worker, Allied Health Professional, spiritual worker, complementary therapist, others);
6. the collaboration aims to provide palliative care for one or more target diagnostic groups in the study (COPD/CHF/Advanced cancer).

### Participants (embedded subunits)

Patients with advanced cancer, COPD or CHF, their family caregivers and professional caregivers will be recruited from each IPC initiative taking part in the study. Signed informed consent forms will be obtained before study entry.

#### Patients and family caregivers

Patients (and if present one family caregiver per patient) will be recruited by their attending doctor or other professional involved in their care. A family caregiver, defined as the person who non-professionally takes care and supports the patient for most of the time, will be identified by the patient. Family caregivers may not necessarily be a family member [30]. Participants need to meet the inclusion criteria (see “Inclusion criteria for patients and family caregivers”) in order to be eligible to take part in the study. In order to gain variation in patients’ experiences with care provision at different time points in their disease trajectories, we strive to purposively recruit patients who are at different time points in their disease trajectories.

### Inclusion criteria for patients and family caregivers

#### Patients & family caregivers

1. 18 years or above
2. Able to communicate in the national language (Dutch, English, German, Hungarian)
3. Cognitively able to complete questionnaires and to participate in interviews.

#### Patients

1. The patient’s attending doctor answers “No” to the surprise question: “Would you be surprised if the patient died within 1 year?”
2. Any of the following diagnoses:
  A) Advanced cancer (cancer with local progression and/or distant metastasis at presentation)
  B) Severe heart failure in accordance with NYHA classification stage III-IV
  C) COPD Gold stage IV classification

We aim to achieve a total sample of 138 included patients and 138 family caregivers. This means an inclusion of six patients and six family caregivers per IPC initiative. We expect that this small number is attainable within 18 months, as this will be done in a multicenter study across 5 European countries with dedicated researchers per site. Herewith, we have also taken into account that the qualitative data analysis is not postponed to the end of data collection, but will already start after the first interviews, as data analysis in qualitative research is an iterative process until the end of the data collection period. In the recruitment of patients and family caregivers we will take account of a 30% attrition rate [31].

#### Professional caregivers

Professional caregivers who are involved in the patient’s care network (identified by the patient) and who are involved in the selected IPC initiatives will be invited for participation in a focus group. We aim to conduct 23 focus groups each with 6-10 participants. In order to maximize an exploration of different perspectives on the initiative as it is experienced in practice, we aim at a convenience sample containing various professional roles and responsibilities within the initiative. The final invitation list will be made after inclusion of the last patient, with alternatives in case of decline.

### Data collection

There will be four consecutive contact moments with patients and family caregivers with an interval of one month (baseline, month 1, month 2, month 3). These are displayed in Table 1. At baseline and at month 3 there will be a face-to-face contact with the patient and his/her family caregiver. During these contacts we will conduct semi-structured interviews and assess the caregiver network analysis with the patient. At baseline, month 1, month 2 and month 3 the patient and family caregiver will complete the questionnaires. Weekly, between baseline and month 3, the patient will keep a diary. The questionnaires and diary will be completed by the patient and/or family caregiver themselves or with the help of a researcher, by telephone.

**Table 1:** **Data collection schedule**

Outcome parameter	Data collection method	Baseline	Week 1-4	Month 1	Week 5-8	Month 2	Week 9-12	Month 3	End of data collection
**Patient**									
Organisation of patient’s care network, Collaboration between professional caregivers	Caregiver network analysis questionnaire	**X**						**X**	
Experiences with IPC initiative, Quality of Care	Semi-structured interview	**X**						**X**	
	Diary		X		X		X		
Quality of Life, Perceived symptoms	Palliative care Outcome Scale	**X**		**X**		**X**		**X**	
Satisfaction with Care	Canhelp Lite	**X**		**X**		**X**		**X**	
**Family caregiver**									
Experiences with IPC initiative, Quality of Care	Semi-structured interview	**X**						**X**	
Quality of Life, Perceived symptoms of patient	Palliative care Outcome Scale	**X**		**X**		**X**		**X**	
Satisfaction with Care	Canhelp Lite	**X**		**X**		**X**		**X**	
Burden & Reward of care giving	Caregiver Reaction Assessment	**X**		**X**		**X**		**X**	
**Professional caregiver**									
Experiences with IPC initiative, Quality of Care, Collaboration between professional caregivers	Focus group								**X**

If a patient dies during the study period, we will contact the family caregiver to offer our condolences and, dependent on the circumstances of the bereaved carer, will ascertain if they wish to have a final semi-structured interview. If so, we will contact them again to make an arrangement for the final interview at a time of their convenience between 4 and 12 weeks afterwards. We will not administer any questionnaires anymore. Per initiative there will be one focus group with professional caregivers at the end of the data collection.

The expected duration of the entire study period will be 18 months, including recruitment, data collection, and analysis. Data collection is scheduled to start in June 2014 and to finish at the end of 2015. The data collection methods that will be applied to assess the outcome parameters are described in Table 1.

### Patients

#### Social Network Analysis

The organisation of the care network of patients, including the type, properties and quality of relationships between patients, family caregivers and professional caregivers will be examined using a social network approach. Social network analysis (SNA) is a method to investigate patterns of relations, communication and collaboration between actors in a given network. In health care research SNA has been applied to investigate organisational structures, processes, and service provision. The results can be used to design or implement interventions to change health care policy or practice [32–34]. To analyse the organisation of the patient’s care network a structured questionnaire, based on previously published questionnaires [35, 36], was developed by the project team. The final questionnaire (see Additional file 1) contains 13 questions and examines contacts between patient and caregivers in his/her care network, evaluation of services provided, perceived continuity of care, and collaboration between caregivers in the network. The patient’s care network, including the type, properties and quality of relationships will be further explored using a patient diary and semi-structured interviews (discussed below).

#### Patient diary

A patient diary will be used to collect data about the perceived quality of care during the palliative care trajectory and the nature of professional caregiver contact. The diary will be kept weekly and contains two questions:

1. Did you have contact with a non-family caregiver during the last week? (e.g. palliative care team consult, home care, GP, psychologist, hospital, other)
2. If yes, how would you rate the care you received?

The answer to the second question is given on a 5-point (Likert) scale, rating from 1 (poor) to 5 (excellent). Information provided in the diary will be explored in the last semi-structured interview. If the patient is unable to fill in the diary on his/her own the family caregiver/researcher may assist the patient.

Recording contacts between patients and their caregivers will provide evidence about care utilisation and perceived quality. Information gleaned from the patient diary and the SNA will enable an in-depth examination of the development and changes in the patient’s care network over time. Combining data from both tools – together with that from the semi-structured interviews (discussed below) – will also allow a detailed explication of the extent of palliative care integration in the participating initiatives.

### Patients and family caregivers

#### Semi-structured interview

Semi-structured interviews will be used to explore views of patients and family caregivers about their experiences with the IPC services they receive. These will be conducted by trained researchers from the project teams in each of the five participating countries. In principle the interviews will be conducted separately. For practical reasons the researcher may deviate from this and conduct the interview with both patient and family caregiver at the same time. In order to minimize influences between patients and family caregivers when they are together, we will emphasize before the start of the interview that we are interested in both the personal view of the patient and the family caregiver. Further we will ask either the patient or family caregiver directly for his/her own view, e.g.: “Is this problem you just mentioned also a problem in your own view, or is this a problem in the view of [name of family caregiver]?”. Patients in this study are vulnerable and often more comfortable in the surrounding of their partner, so we do not want to be too strict in a separate interview to restrict the burden for the patient as much as possible. Topics of the interviews include:

1. Exploration of problems and needs of the patient
2. Exploration of the contacts and relationships of patients and family caregivers with professional caregivers
3. Exploration of satisfaction and perceived deficits in service provision from the perspective of patients and family caregivers
4. Exploration of the views of patients and family caregivers on the collaboration between professional caregivers in the care network of the patient. A second interview at month 3 will enable an exploration of the care experience over time.
5. The final interview will include a review on the most important problems and needs in the dying phase from the perspective of the bereaved family caregiver, which caregivers were involved in this phase and just after bereavement, and how the care provision was experienced by the bereaved family caregiver.

### Questionnaires

Demographic and other relevant data that describe the population contributing to this study will be collected at baseline using the questionnaires presented in Table 1. With regard to the vulnerable population of seriously ill patients and the international nature of the study, questionnaires were selected based on: validation and/or applicability in palliative care populations of patients with cancer and chronic disease, time needed for completion in order to limit burden of assessment, and available translations into the national languages of the countries involved in the study (Dutch, English, German, Hungarian). Questionnaires that had not yet been translated were translated using a forward-backward translation procedure [37].

#### Palliative care Outcome Scale (POS)

The Palliative care Outcome Scale – version 1 will be used with patients and family caregivers to measure quality of life and perceived symptoms of patients. It is widely used and tested and is validated for use in palliative care. The completion time is short, approximately 7 minutes [38]. The POS has been translated into Chinese, English, Dutch, German, Italian, Portuguese, Punjabi, Spanish and Urdu. There is a patient and a caregiver version.

#### Canhelp Lite

The Canhelp Lite will be used with patients and family caregivers to measure satisfaction of care. It was developed in Canada, validated for use in palliative care and applied to patients with advanced, life-limiting illnesses. It is applicable in both institutional and community based settings. There is a Patient Questionnaire, Caregiver Questionnaire and a Bereavement Questionnaire [39]. Only the Patient Questionnaire and Caregiver Questionnaire will be used in this study. The Canhelp Lite has a completion time of approximately 10 minutes for both the patient and caregiver version and has been translated into English and French.

### Family caregivers

#### Caregiver Reaction Assessment (CRA)

The Caregiver Reaction Assessment will be used with family caregivers to measure their perceived burden and reward of care giving. It measures both positive and negative reactions to care giving [40]. The CRA is widely used and extensively tested and has a completion time of approximately 10 minutes. It has been translated into Dutch, English, German, Japanese, Norwegian and Thai. The CRA has been applied to family caregivers as well as significant others for patients with physical, chronic and mental impairments and malignant diseases [41].

### Professional caregivers

#### Focus group

Focus groups will be used to obtain insight into the experiences of professional caregivers with providing IPC. The interviews will address professional caregivers’ views concerning the quality of IPC in their initiative and issues involved in working across organisational boundaries to provide that care. One focus group will be conducted in each of the participating services. Topics that will be discussed include: components considered important for high quality integrated care, set-up of the initiative (roles, responsibilities, relationships), expectations and/or future improvements.

### Data management and analysis

Anonymous participant data will be stored in a protected database Castor EDC (Electronic Data Collection) with a login function. The master database will be kept at the centre of the research coordinator Radboud University Medical Centre in the Netherlands.

Interviews will be tape recorded and transcribed verbatim. Transcriptions will be analysed using content analysis techniques supported by the qualitative analysis software package NVivo 10. Researchers from each partner country will jointly develop a preliminary coding schedule with the results of the first two interviews. This code book will be used for the baseline and final interviews. The codes and themes will form the basis of the coding strategy throughout data collection and the data analysis. Analysis will be iterative during the fieldwork phase in order to allow emergent themes to be incorporated into the data collection. This procedure will also be used to analyse the focus group data. For the focus groups a separate code book will be developed by the researchers.

In general, the analysis will focus on the similarities and differences between the IPC initiatives (e.g. the diagnostic group(s), organisational structure, the caregivers and settings that are involved in the care provision) and what these mean for the views of patients, family caregivers and professional caregivers on the care provision. The analysis will result in five country specific reports about the experience of using and providing IPC in the last year of life. IPC country specific reports will be analysed and summarised in one overarching document. This report will provide a trans-national perspective on the lived experience of IPC services from the particular standpoints of the service users and professional caregivers involved.

Statistical analysis will be performed using SPSS Statistics 20 on the quantitative data derived from the questionnaires. We will use descriptive statistics such as frequencies, crosstabs, means, standard deviation in the data analysis in order to describe the characteristics of the participant population. During this analytical phase we will integrate the quantitative variables and qualitative findings so as to draw a more complete picture of IPC across Europe.

### Training sessions

In order to assure high quality and uniformity of data collection and analysis in all five countries two training sessions will be organised. These sessions will support researchers in preparing and conducting interviews, performing reliable and valid qualitative research and processing data. Training will also focus on preparing researchers to conduct research with potentially vulnerable participants.

### Ethical issues

As in all ethically conducted research, informed consent will be obtained to guarantee voluntary participation and participants may withdraw at any time should they wish to. For these reasons we believe that the potential for risk in this study is minimal and that it may even benefit participating patients and family caregivers [42, 43].

In order to test this expectation we added four questions about how patients experienced participation in the interview study (“thoughts on the studies”) and potential distress or satisfaction related to the study participation. These questions will be asked after the first and final patient interview. The questions were derived from Gysels [42] who conducted a qualitative study with 76 palliative care patients from the UK. This study concluded that although patients experienced thinking about the future as difficult, sharing problems was therapeutic and being able to contribute to research was considered empowering [42]. Using the same questions in our study enables us to contribute to this ethical debate.

In order to minimize the burden of data collection on patients and family caregivers, questionnaires and diaries have a short completion time and the total duration of interviews will be limited to a maximum of 60 minutes. Interviews will be conducted by researchers who are experienced and well trained in research with vulnerable patients.

For professional caregivers, participating in the study may be beneficial because during the interviews they will have the opportunity to reflect on their experiences of the provision of palliative care and their collaboration with other professional caregivers involved in the IPC initiative under examination. This reflection could be a prompt to improve their collaboration with other caregivers and may provide an impetus to improve daily practice in their local collaborations.

Ethical approval has been granted by the ethical review committees of Hungary, The United Kingdom and Germany. The study does not fall within the remit of the Dutch Act on Human Research and for this reason did not have to go through the Dutch ethical review committee. In Belgium the ethical review procedure is in a final stage.

## Discussion

### Strengths

The prospective multiple embedded case study design allows for exploration of IPC trajectories as experienced by patients and family caregivers. This includes palliative care service utilisation, perceived quality of life, quality of care, symptoms and perspectives on the collaboration between caregivers in the patient’s care network. The prospective design allows the possibility to examine these palliative care trajectories more in-depth. It also enables the exploration of IPC services utilisation and the collaboration between caregivers within the patient’s care network throughout the palliative care trajectory over time.

The international perspective of this study has the advantage that we can compare experiences of service users and providers in a range of different health care contexts in Europe. We expect that this will provide valuable information about barriers, opportunities and good practices in palliative care integration in the selected initiatives across countries. This information can be used in the benchmarking of initiatives in Europe and the further implementation of integrated care.

### Challenges

One challenge of a patient study on an international scale is that it needs to meet ethical requirements in several countries. Our study shows that it is feasible to develop such an international multicenter palliative care patient study protocol which meets the nuanced requirements of different national ethical and research governance processes, whilst applying the same data collection and scientific analysis procedures across national boundaries.

We realise that three months is a rather arbitrary period to follow patients who may have a much longer or shorter palliative care trajectory. Identifying those who are in the last year of life, yet functioning well enough to engage with and complete the study over a three month period can be problematic. This challenges us to collect as complete information as possible about experiences of patients throughout their entire palliative care trajectory and to warrant accurate inclusion of patients.

## Conclusion

This study will provide valuable data about patients’, family and professional caregivers’ experiences with various IPC initiatives, including quality of care, quality of life, symptoms, burden and reward of care giving, relationships with and collaboration between professional caregivers. These data will provide important insights into what constitutes best practice, as perceived by those using and providing IPC services, across a range of different health economies in Europe. This knowledge will add to the scientific evidence for IPC services internationally and will contribute to improvements in the quality of care and the quality of living and dying of severely ill patients and their relatives in Europe.

## Electronic supplementary material

Additional file 1: **Caregiver network analysis questionnaire.** Presents the questions used in the caregiver network analysis questionnaire. (PDF 72 KB)
