# Supplementary material for: Experiences of patients, family and professional caregivers with Integrated Palliative Care in Europe: protocol for an international, multicenter, prospective, mixed method study
Source: BMC Palliat Care. 2014 Nov 21;13:52. doi: 10.1186/1472-684X-13-52 (PMC4254197; doi:10.1186/1472-684X-13-52)
Supplement: Supplementary file 1 — Additional file 1: Caregiver network analysis questionnaire. Presents the questions used in the caregiver network analysis questionnaire. (PDF 72 KB) [file 12904_2014_233_MOESM1_ESM.pdf]

Additional file 1: Caregiver network analysis questionnaire – patient

1. **Which caregivers do you have contact with to receive daily care, advice about your disease, medical treatment, psychological support, finances, etc.?**

**Family/informal caregiver**	☐ Yes	☐ No
**GP**	☐ Yes	☐ No
**Home care worker**	☐ Yes	☐ No
**Nurse**	☐ Yes	☐ No
**Medical specialist**	☐ Yes	☐ No
**Allied Health Professional**	☐ Yes	☐ No
**Social Worker**	☐ Yes	☐ No
**Faith leader**	☐ Yes	☐ No
**Psychological support worker**	☐ Yes	☐ No
**Hospice worker**	☐ Yes	☐ No
**Other, …**	☐ Yes	☐ No

2. **For those with whom you have contact, how often and how do you have contact?**

**Family/informal caregiver**	☐Daily ☐Weekly ☐Monthly ☐Less Often	☐ Face-To-Face ☐ Telephone ☐ E-Mail
**GP**	☐Daily ☐Weekly ☐Monthly ☐Less Often	☐ Face-To-Face ☐ Telephone ☐ E-Mail
**Home care worker**	☐Daily ☐Weekly ☐Monthly ☐Less Often	☐ Face-To-Face ☐ Telephone ☐ E-Mail
**Nurse**	☐Daily ☐Weekly ☐Monthly ☐Less Often	☐ Face-To-Face ☐ Telephone ☐ E-Mail
**Medical specialist**	☐Daily ☐Weekly ☐Monthly ☐Less Often	☐ Face-To-Face ☐ Telephone ☐ E-Mail
**Physiotherapist**	☐Daily ☐Weekly ☐Monthly ☐Less Often	☐ Face-To-Face ☐ Telephone ☐ E-Mail
**Social Worker**	☐Daily ☐Weekly ☐Monthly ☐Less Often	☐ Face-To-Face ☐ Telephone ☐ E-Mail
**Faith leader**	☐Daily ☐Weekly ☐Monthly ☐Less Often	☐ Face-To-Face ☐ Telephone ☐ E-Mail
**Psychological support worker**	☐Daily ☐Weekly ☐Monthly ☐Less Often	☐ Face-To-Face ☐ Telephone ☐ E-Mail
**Hospice worker**	☐Daily ☐Weekly ☐Monthly ☐Less Often	☐ Face-To-Face ☐ Telephone ☐ E-Mail
**Other, …**	☐Daily ☐Weekly ☐Monthly ☐Less Often	☐ Face-To-Face ☐ Telephone ☐ E-Mail

3. **For those with whom you have contact, how would you rate your relationship with this caregiver?**

**Family/informal caregiver**	☐Excellent	☐Very Good	☐ Good	☐Reasonable	☐Poor
**GP**	☐Excellent	☐Very Good	☐ Good	☐Reasonable	☐Poor
**Home care worker**	☐Excellent	☐Very Good	☐ Good	☐Reasonable	☐Poor
**Nurse**	☐Excellent	☐Very Good	☐ Good	☐Reasonable	☐Poor
**Medical specialist**	☐Excellent	☐Very Good	☐ Good	☐Reasonable	☐Poor
**Physiotherapist**	☐Excellent	☐Very Good	☐ Good	☐Reasonable	☐Poor
**Social Worker**	☐Excellent	☐Very Good	☐ Good	☐Reasonable	☐Poor
**Faith leader**	☐Excellent	☐Very Good	☐ Good	☐Reasonable	☐Poor
**Psychological support worker**	☐Excellent	☐Very Good	☐ Good	☐Reasonable	☐Poor
**Hospice worker**	☐Excellent	☐Very Good	☐ Good	☐Reasonable	☐Poor
**Other, …**	☐Excellent	☐Very Good	☐ Good	☐Reasonable	☐Poor

4. **For those with whom you have contact, how would you rate care that you receive from these caregiver?**

**Family/informal caregiver**	☐Excellent	☐Very Good	☐ Good	☐Reasonable	☐Poor
**GP**	☐Excellent	☐Very Good	☐ Good	☐Reasonable	☐Poor
**Home care worker**	☐Excellent	☐Very Good	☐ Good	☐Reasonable	☐Poor
**Nurse**	☐Excellent	☐Very Good	☐ Good	☐Reasonable	☐Poor
**Medical specialist**	☐Excellent	☐Very Good	☐ Good	☐Reasonable	☐Poor
**Physiotherapist**	☐Excellent	☐Very Good	☐ Good	☐Reasonable	☐Poor
**Social Worker**	☐Excellent	☐Very Good	☐ Good	☐Reasonable	☐Poor
**Faith leader**	☐Excellent	☐Very Good	☐ Good	☐Reasonable	☐Poor
**Psychological support worker**	☐Excellent	☐Very Good	☐ Good	☐Reasonable	☐Poor
**Hospice worker**	☐Excellent	☐Very Good	☐ Good	☐Reasonable	☐Poor
**Other, …**	☐Excellent	☐Very Good	☐ Good	☐Reasonable	☐Poor

Statements and questions about collaboration between caregivers from whom you receive care:

5. **These caregivers appear to work together very well.**

   ☐Strongly Agree ☐Agree ☐Neutral ☐Disagree ☐Strongly Disagree

6. **The care given by these caregivers appears to be well-connected.**

   ☐Strongly Agree ☐Agree ☐Neutral ☐Disagree ☐Strongly Disagree

7. **These caregivers pass on information to each other well.**

   ☐Strongly Agree ☐Agree ☐Neutral ☐Disagree ☐Strongly Disagree

8. **These caregivers always know very well what the other caregivers have done.**

   ☐Strongly Agree ☐Agree ☐Neutral ☐Disagree ☐Strongly Disagree

9. **Sometimes I perceive friction between caregivers.**

   ☐Strongly Agree ☐Agree ☐Neutral ☐Disagree ☐Strongly Disagree

10. **Who, do you think, is the main responsible caregiver of all caregivers you receive care from?** (i.e. the person(s) who decide(s) how your care is being organised)

11. **Is there anything missing from the care you receive and who do you think should be providing it?** (Which persons/organisations do you miss and why?)

12. **In your opinion, might there be too many caregivers from whom you receive care?** (Which persons/organisations may be redundant and why?)
